# Dosing and treatment duration of suppressive antimicrobial therapy in orthopedic implant infections: a cohort study

**DOI:** 10.5194/jbji-9-149-2024

**Published:** 2024-06-04

**Authors:** Jaap L. J. Hanssen, Robert J. P. van der Wal, Henrica M. J. van der Linden, Joffrey van Prehn, Henk Scheper, Mark G. J. de Boer

**Affiliations:** 1 Leiden University Center for Infectious Diseases (LU-CID), Infectious Diseases, Leiden University Medical Center, Leiden, the Netherlands; 2 Department of Orthopedic Surgery, Leiden University Medical Center, Leiden, the Netherlands; 3 Leiden University Center for Infectious Diseases (LU-CID), Medical Microbiology and Infection Control, Leiden University Medical Center, Leiden, the Netherlands; 4 Department of Clinical Epidemiology, Leiden University Medical Center, Leiden, the Netherlands

## Abstract

**Introduction**: Limited data inform about the optimal dosing and duration of suppressive antimicrobial therapy (SAT) for orthopedic implant infection (OII). We aimed to compare the effectiveness of low-dosage with standard-dosage SAT and evaluate the safety of stopping SAT. **Methods**: All patients with OII treated with SAT from 2011 to 2022 were retrospectively included. Data were extracted from electronic patient files. Low-dosage SAT was defined as antimicrobial therapy dosed lower than the standard dosage recommended for OII. The association of dosing strategy and other factors with failure-free survival were assessed by Kaplan–Meier and Cox proportional hazard models. **Results**: One-hundred-and-eight patients were included. The median follow-up time after SAT initiation was 21 months (interquartile range (IQR) 10–42 months). SAT was successful in 74 patients (69 %). Low-dosage SAT ( n=82 ) was not associated with failure in univariate (hazard ratio (HR) 1.23, 95 % confidence interval (CI) 0.53–2.83) and multivariate analyses (HR 1.24, 95 % CI 0.54–2.90). In 25 patients (23 %), SAT was stopped after a median treatment duration of 26 months. In this group, one patient (4 %) developed a relapse. **Conclusions**: In this study, low-dosage SAT was as effective as standard dosage SAT. Moreover, stopping SAT after 2 to 3 years may be justified in patients with a good clinical course. These findings warrant further research on optimal dosing and duration of SAT and on the durability of in vivo biofilms.

## 1 Introduction

Patients with orthopedic implant infection (OII) managed with implant retention and a high likelihood of relapse after initial treatment often receive chronic suppressive antimicrobial therapy (SAT). Reported success rates of this strategy vary between 23 % and 95 %, and the preferred regimen, dosage and treatment duration of SAT differ around the world (Cobo and Escudero-Sanchez, 2021).

With respect to the dosage, the 2013 Infectious Diseases Society of America (IDSA) guideline suggests that antibiotics can be lowered for SAT compared to the standard prosthetic joint infection (PJI) treatment dosages (Osmon et al., 2013). Studies specifically addressing low-dosage SAT have not been published, although low-dosage SAT is mentioned in several studies (Siqueira et al., 2015; Bryan et al., 2017; Prendki et al., 2017; Wouthuyzen-Bakker et al., 2017).

Another important clinical question is whether SAT can ever be discontinued and, if so, on which clinical criteria. The IDSA guideline recommends indefinite oral SAT in patients with PJI who qualify for SAT (Osmon et al., 2013). Most studies on SAT for PJI also reported the duration of suppression to be lifelong (Prendki et al., 2017; Wouthuyzen-Bakker et al., 2017; Pradier et al., 2018; Escudero-Sanchez et al., 2020).

Over the years, it became common practice in our hospital to treat patients on SAT with a dosage that is lower than the standard (i.e. therapeutic) dosage used in the treatment of OII. This was believed to be a reasonable approach due to the presumed low bacterial load after the initial antibiotic treatment period combined with our clinical experience that lowering the dosage of SAT in patients (due to side effects) did not result in more relapses. Further, SAT was increasingly being stopped after 2 to 3 years in patients with good clinical performance rather than continuing with suppression indefinitely. The aim of this study was to analyze the clinical outcomes of this treatment strategy for OII.

## 2 Methods

### 2.1 Study design and population 

This retrospective observational study was conducted in a tertiary care hospital in the Netherlands. The inclusion period ranged from 1 June 2011 to 1 November 2022. All consecutive patients with PJI, fracture-related infection (FRI) and spinal implant infection (SII) who started on SAT were eligible for inclusion. These eligible participants were identified using CTcue text-mining software (IQVIA B.V., Amsterdam, the Netherlands). Clinical data were manually extracted after patient file review by a single researcher (Jaap L. J. Hanssen), who consulted one of the senior co-authors (Mark G. J. de Boer or Henk Scheper) when in doubt of a case. Henk Scheper validated data from a random set of cases (10 % of total). Exclusion criteria were age < 16 years, follow-up less than 1 month from start of SAT and unlikely PJI according to the European Bone and Joint Infection Society (EBJIS) criteria (McNally et al., 2021). Follow-up time started on the first day of SAT.

### 2.2 Standard management of OII

Since 2015, a multidisciplinary team (MDT) was implemented, and team members discussed all patients with OII during weekly meetings. Team members were orthopedic and trauma surgeons, infectious disease specialists, and clinical microbiologists. Standard management of OII consisted of debridement, antibiotics and implant retention (DAIR) for acute OII and revision surgery in combination with antibiotics for chronic OII, unless specific conditions dictated otherwise. All study participants were treated with surgical debridement unless a contra-indication for surgery existed or if the patient refused surgery. Antimicrobial treatment consisted of 1 to 2 weeks of intravenous (IV) antibiotics followed by 4 to 11 weeks of targeted oral antimicrobial therapy (for a total duration of 6 to 12 weeks). The decision to start SAT after the initial therapeutic antimicrobial treatment episode was made by the MDT and based on the resolution of symptoms and normalization of inflammatory parameters. Besides the scheduled cessation of SAT in the case of good clinical performance, suppression could also be stopped if unacceptable side effects to antibiotics arose or if this was requested by the patient.

### 2.3 Study definitions

PJI and FRI were defined according to the 2021 EBJIS definitions (Govaert et al., 2020; McNally et al., 2021). The PJI criteria were also applied to SII due to a lack of specific diagnostic criteria for SII.

For this study, SAT was defined as prolonged oral antimicrobial therapy after the initial standard treatment of 6 to 12 weeks. Low-dosage SAT was defined as antimicrobial treatment that was lower or less frequently dosed than the oral therapeutic dosage (standard-dosage SAT) recommended for the treatment of OII in our hospital. The dosages used in this study are summarized in Table 1.

**Table 1:** Dosing schedules of suppressive antimicrobial therapy in the standard-dosage group and in the low-dosage group in this study.

	Standard-dosage SAT	Low-dosage SAT
Amoxicillin	1000 mg t.i.d. or q.i.d.	500 mg b.i.d., t.i.d. or q.i.d. 1000 mg b.i.d
Flucloxacillin	1000 mg q.i.d.	500 mg b.i.d, t.i.d. or q.i.d. 1000 mg b.i.d. or t.i.d.
Amoxicillin / clavulanic avid	1250 mg t.i.d.	625 mg b.i.d
Pheneticillin	1000 mg q.i.d.	500 mg q.i.d.
Ciprofloxacin	500–750 mg b.i.d.	500–750 mg q.d.
Levofloxacin	500 mg b.i.d.	250–500 mg q.d.
Moxifloxacin	400 mg q.d.	–
Clindamycin	600 mg t.i.d.	300 mg b.i.d. or t.i.d. 600 mg b.i.d.
Trimethoprim / sulfamethoxazole	960 mg b.i.d.	480 mg q.d or b.i.d. 960 mg q.d
Doxycycline	100 mg b.i.d.	100 mg q.d.
Linezolid	600 mg b.i.d.	150–600 mg q.d.
Rifampicin^*^	450–600 mg b.i.d.	300 mg q.d.
Fluconazole	200 mg b.i.d.	200 mg q.d.

The abbreviations used in the table are as follows: SAT – suppressive antimicrobial therapy; q.d. – once daily; b.i.d. – twice daily; t.i.d. – three times a day; q.i.d. – four times a day. ^*^ In combination with levofloxacin.

Patients were categorized into two groups: a low-dosage group and a standard dosage group.

OIIs were classified as early postoperative (less than 3 months after surgery), late chronic (symptoms more than 3 weeks and diagnosis more than 3 months after surgery) and acute hematogenous (symptoms less than 3 weeks in a previously asymptomatic patient at least 3 months after surgery).

For the purpose of this study, we retrospectively defined two indications for SAT. The first was (i) “certain” relapse (without SAT) – meaning OII treated without any surgery, late chronic infection treated with DAIR or acute infection with failure of DAIR – and the second was (ii) “high risk” of relapse (without SAT) – meaning early postoperative and acute hematogenous OII treated with DAIR in the presence of at least one of the following risk factors for relapse: tumor endoprosthesis, previous failures, poor soft tissue and/or bone stock, significant comorbidity (e.g. on chemotherapy, active rheumatoid arthritis), and difficult-to-treat microorganisms (e.g. *Candida albicans*) (Cobo and Escudero-Sanchez, 2021).

Failure was defined as one of the following outcomes: the appearance or persistence of a fistula, unplanned surgical intervention or admission for IV antibiotics, increasing the low-dosage SAT to standard dosage, restart of antimicrobial treatment after stopping SAT, uncontrolled symptoms, or death related to the infection. SAT was considered successful if none of these events occurred. Endpoints were failure, death unrelated to OII or latest follow-up at the outpatient clinic when no event occurred.

### 2.4 Statistical analysis 

Continuous variables were described as means with 95 % confidence intervals (CI) or as medians with interquartile ranges (IQR). Normally distributed data were compared between groups using Student's t test, and non-normally distributed data were compared with a Mann–Whitney U test. Categorical variables were compared with the chi-square test or with Fisher's exact test if more than 20 % of cells had expected frequencies of less than five. The primary outcome was treatment failure-free survival time, assessed by Kaplan–Meier analysis. Patients who died due to a cause not related to the OII or who underwent a planned removal of their implant in the case of FRI and SII were censored at the time of this event. SAT dosing and other factors potentially associated with failure were assessed by Cox proportional hazard models. Variables were considered for multivariable analysis in the case of p < 0.10 in univariable analysis. SPSS Statistics for Windows was used (IBM SPSS Statistics for Windows, Version 29.0.0.0, Armonk, NY).

## 3 Results

During the study period, 113 patients were eligible for inclusion. Five patients were excluded: two patients were lost to follow-up within 1 month, two patients died within 2 weeks after initiation of SAT because of metastasized cancer, and in one patient PJI was unlikely based on the EBJIS 2021 criteria. The baseline characteristics of all 108 included patients are presented in Table 2.

**Table 2:** Baseline clinical characteristics of all 108 patients with suppressive antimicrobial therapy.

	All patients	Low-dosage SAT	Standard-dosage SAT	p value
	n = 108	n = 82	n = 26	
Age at diagnosis (median, IQR)	65 (50–73)	65.5 (50.8–74)	65.5 (36.3–72.3)	0.53
Male sex ( n , %)	60 (56)	44 (54)	16 (62)	0.48
Comorbidities				
Smoker	23 (21)	18 (22)	5 (19)	0.75
BMI (mean, 95 % CI)	26.5 (19–36.6)	26.5 (11.8–41.6)	26.8 (12.9–40.7)	0.82
Charlson Comorbidity Index (median, IQR)	3 (2–5)	3 (2–5)	3 (2–5)	0.80
Rheumatoid arthritis	9 (8)	7 (9)	2 (8)	1.00
Sarcoma	28 (26)	16 (20)	12 (46)	0.01
Chemotherapy	8 (7)	7 (9)	1 (4)	0.68
Type of implant				0.11^a^
Prosthetic joint	67 (62)	47 (57)	20 (77)	
Tumor endoprosthesis^b^	38 (35)	25 (31)	13 (50)	
Osteosynthesis	24 (22)	19 (23)	5 (19)	
Spinal implant	17 (16)	16 (20)	1 (4)	
Implant site				0.50^a^
Hip	35 (32)	23 (28)	12 (46)	
Knee	29 (27)	22 (27)	7 (27)	
Upper limb	13 (14)	11 (13)	2 (8)	
Revised implant	43 (40)	33 (40)	10 (39)	0.87
Previous OII in the same joint	37 (34)	30 (37)	7 (27)	0.37
EBJIS 2021 criteria^c^				0.07^a^
Confirmed infection	86 (79)	62 (76)	24 (92)	
Suggestive/likely infection	22 (17)	20 (24)	2 (8)	
Timing infection				0.56^a^
Early postoperative	57 (53)	41 (50)	16 (62)	
Acute hematogenous	13 (12)	10 (12)	3 (12)	
Late chronic	38 (35)	31 (38)	7 (27)	
C-reactive protein at diagnosis in mg L^-1^ (median, IQR)	76 (30–172)	73 (29–194)	103 (41–141)	0.62
C-reactive protein in mg L^-1^ at start of SAT	10 (5–21)	10 (5–22)	9 (3–20)	0.57
Weeks of antibiotic treatment before SAT (median, IQR)	8 (6–13)	8 (6–13.3)	9 (6.8–13.3)	0.60
Indication for SAT				0.58^a^
Certain failure^d^	55 (51)	43 (52)	12 (46)	
High risk of failure^e^	53 (49)	39 (48)	14 (54)	
Microorganisms				0.11^a^
*Staphylococcus aureus*	35 (32)	29 (35)	6 (23)	
Coagulase-negative staphylococci	34 (32)	29 (35)	5 (19)	
Gram-negatives	26 (24)	15 (18)	11 (42)	
Enterococci	23 (21)	14 (17)	9 (35)	
Streptococci	19 (18)	15 (18)	4 (15)	
*Cutibacterium acnes* ^f^	14 (14)	12 (15)	2 (8)	
Anaerobes	10 (9)	9 (11)	1 (4)	
*Candida albicans*	3 (3)	1 (1)	2 (8)	
Corynebacteriaceae	3 (3)	3 (4)	0	
Polymicrobial infection	42 (39)	30 (37)	12 (46)	0.38

The abbreviations used in the table are as follows: SAT – suppressive antimicrobial therapy; OII – orthopedic implant infection; CI – confidence interval; IQR – interquartile range. ^a^ chi-square test for distribution of categorical variables. ^b^ subgroup of prosthetic joint. ^c^ according to EBJIS 2021 criteria for PJI and the AO Foundation and EBJIS 2020 consensus definition for FRI. ^d^ OII treated without any surgery, late chronic infection treated with DAIR or acute infection with failure of DAIR. ^e^ early postoperative and acute hematogenous OII treated with DAIR in the presence of risk factors for relapse. ^f^ formerly named *Propionibacterium acnes.*

Indications for SAT in patients with an acute PJI treated with DAIR ( n = 27) were the presence of a tumor endoprosthesis ( n = 18, 67 %), microorganisms associated with higher risk of relapse ( n = 14, 52 %), comorbidity ( n = 9, 33 %) or previous PJI treatment failures ( n = 9, 33 %). Reasons for not performing any surgery in 19 patients were comorbidity ( n = 10, 53 %) (metastasized cancer, chemotherapy and short life expectancy, chronic obstructive pulmonary disease (COPD), heart failure), surgery related factors ( n = 6, 16 %) (poor bone stock, soft tissue problems, prosthesis too complex to remove, non-consolidation of fracture, risks surgery disproportionate to the symptoms) or refusal by the patient ( n = 6, 30 %). The diagnostic criteria for the patients who did not receive any surgery are summarized in Table S1 in the Supplement.

**Figure 1:**
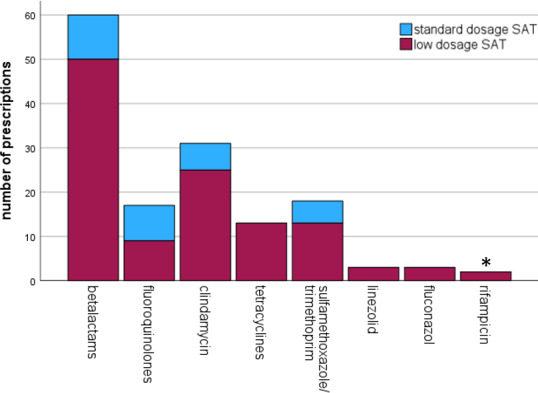
Frequency of antibiotic use for oral suppressive antimicrobial therapy (SAT). ^*^ in combination with levofloxacin.

The number of antibiotics that were used in this study are summarized in Fig. 1. More details regarding the dosing schedules are provided in Table S2.

### 3.1 Clinical outcomes

**Figure 2:**
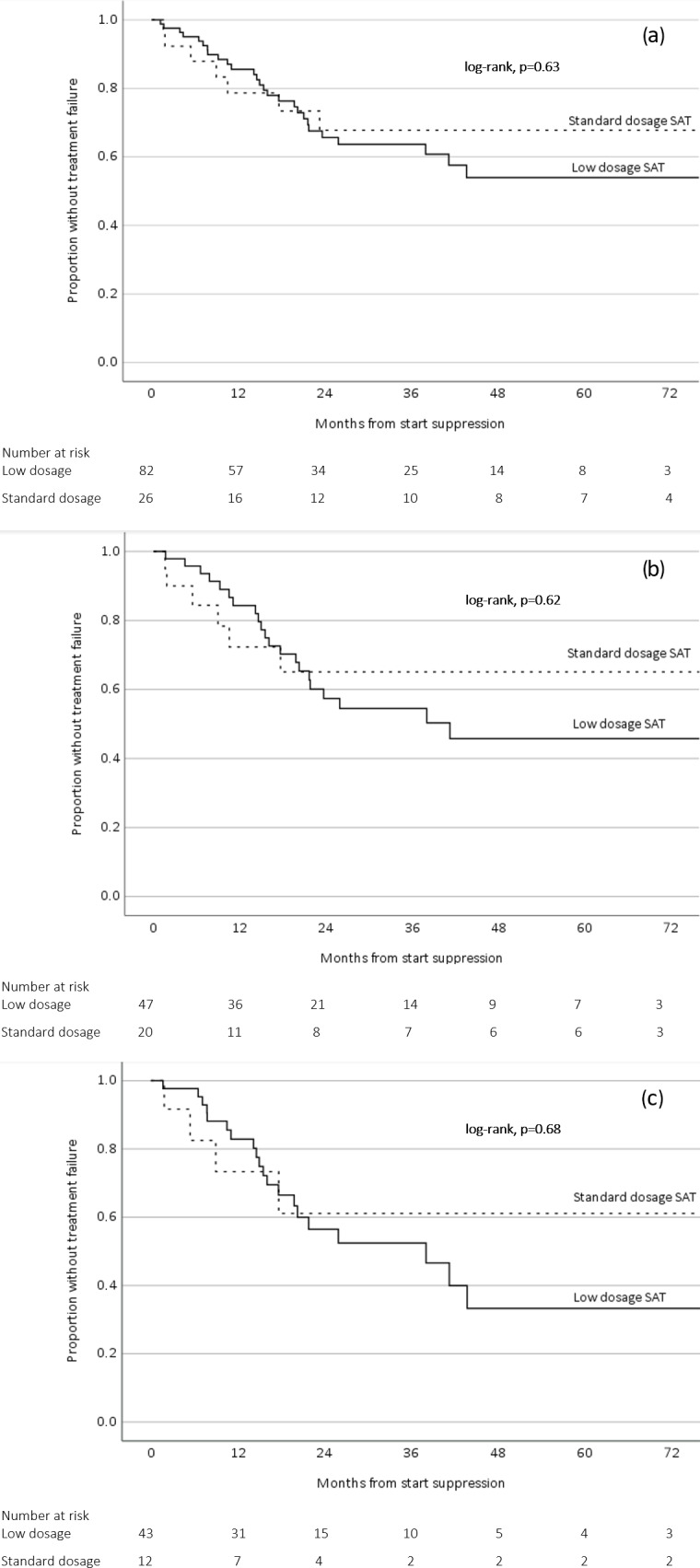
Survival analysis related to low-dosage and standard-dosage SAT **(a)** including all orthopedic implant infections, **(b)** including only prosthetic joint infections, and **(c)** including only orthopedic implant infections with an indication for SAT because of certain relapse.

SAT was considered successful in 74/108 patients (69 %) with a median follow-up of 21.2 months (IQR 10.4–41.8 months). The success rates for patients with PJI, FRI, and SII were 60 %, 88 %, and 79 %, respectively. The SAT failure-free survival in the low-dosage group was lower compared to the standard-dosage group, but this difference was not statistically significant ( p=0.63 ) (Fig. 2a). This outcome did not change when including only patients with PJI (Fig. 2b) or only patients from the group that had an indication for SAT because of “certain” relapse (if SAT would have been withheld) (Fig. 2c).

SAT was discontinued in 25 patients after a median time of 26.4 months (IQR 14.3–38.1 months). This group consisted of 12 PJI (48 %), 9 FRI (36 %) and 4 SII (15 %). Eighteen of these patients (72 %) had a confirmed infection at the time of diagnosis. Five patients (20 %) did not undergo any form of surgery. The median C-reactive protein at the start SAT was 8 mg L^-1^ (IQR 3–13 mg L^-1^). SAT was stopped because of good clinical performance in 23 patients (92 %) and requested by the patient due to side effects in two cases (8 %). The median follow-up duration of this group after discontinuation of SAT was 21 months (IQR 9.4–34.6 months). During this period, one patient with PJI (4 %) developed a culture-negative relapse. This occurred within 1 week after stopping SAT, which the patient had received for 38 months.

Of the 83 patients still on SAT at the end of the study, 23 patients had a follow-up beyond 2 years, of which two patients (9 %) had a relapse (after 41 and 44 months).

The details of the 34 patients with failure are summarized in Table 3. The median time to failure was 11.1 months (IQR 6.5–19.8 months). An overview of the cultured microorganisms in failed cases and details of the development of antimicrobial resistance are summarized in Tables S3, S4 and S5. Fifteen of 108 patients (14 %) died due to non-OII related causes.

**Table 3:** Characteristics of all patients with failure treated with suppressive antimicrobial therapy.

	All patients	Low-dosage SAT	Standard-dosage SAT
	n = 34	n = 27	n = 7
Clinical outcome			
New surgery of infected joint, n (%)	18 (53)	12 (44)	6 (86)
Admission for IV antibiotics	3 (9)	3 (11)	0
Uncontrolled symptoms	4 (12)	3 (11)	1 (17)
Fistula	6 (18)	6 (22)	0
Increasing SAT to standard dosage	2 (6)	2 (7)	0
Relapse after stopping SAT	1 (3)	1(4)	0
Microbiological finding at time of failure			
Relapse with index pathogen	11 (32)	8 (30)	3 (43)
Development of SAT resistance	4 (12)	4 (15)	0
New infection with different pathogen	9 (26)	8 (30)	1 (14)
Culture negative	7 (21)	5 (19)	2 (29)
No tissue for cultures obtained	7 (21)	6 (22)	1 (14)

The abbreviations used in the table are as follows: SAT – suppressive antimicrobial therapy.

Upper limb OII, diabetes mellitus, OII of a revised implant and the “certain” relapse group were associated with failure in the univariable analysis but not in the multivariable analysis (Table 4). After selecting only patients with PJI in the analysis, upper limb PJI (HR 4.41, 95 % CI 1.41–13.76) and diabetes mellitus (HR 3.95, 95 % CI 1.43–10.92) were independently associated with failure (Table S6).

**Table 4:** Analysis of clinical characteristics potentially associated with failure of suppressive antimicrobial therapy for all 108 patients.

	Failure	Univariable analysis	p value	Multivariable analysis	p value
	n (%)	HR (95 % CI)		HR (95 % CI)	
Patient factors					
Age > 70	14 (40)	1.33 (0.67–2.64)	0.41		
Smoker	9 (39)	1.92 (0.89–4.12)	0.10		
Charlson Comorbidity Index > 2	24 (36)	1.33 (0.63–2.78)	0.46		
Diabetes mellitus	5 (56)	3.89 (1.48–10.2)	0.01		
Implant					
Prosthetic joint	27 (40)	1^*^			
Fracture related	5 (21)	0.53 (0.20–1.36)	0.19		
Spinal implant	2 (12)	0.32 (0.08–1.35)	0.12		
Previous implant infection	17 (46)	1.79 (0.91–3.51)	0.09		
Revised implant	21 (49)	2.17 (1.09–4.33)	0.03	2.10 (0.74–3.41)	0.23
Chronic OII	22 (42)	1.93 (0.96–3.9)	0.07		
Tumor endoprosthesis	15 (40)	1.40 (0.71–2.76)	0.33		
Anatomic location					
Hip	9 (26)	1^*^			
Knee	13 (45)	2.13 (0.91–4.99)	0.08		
Upper limb	7 (54)	3.17 (1.16–8.63)	0.02	2.10 (0.90–4.91)	0.09
Microbiology					
*Staphylococcus aureus*	8 (23)	0.76 (0.34–1.66)	0.47		
Coagulase-negative staphylococci	14 (41)	1.59 (0.8–3.15)	0.19		
Streptococci	3 (16)	0.33 (0.1–1.09)	0.07		
Enterococci	8 (35)	1.21 (0.55–2.68)	0.64		
Gram-negatives	12 (46)	1.22 (0.58–2.56)	0.62		
Polymicrobial infection	13 (31)	1.03 (0.52–2.06)	0.93		
Clinical aspects					
< 12 weeks antibiotic treatment before SAT	23 (30)	0.79 (0.40–1.58)	0.51		
C-reactive protein at start of SAT > = 20	10 (37)	1.83 (0.86–3.85)	0.12		
Low-dosage SAT	27 (33)	1.23 (0.53–2.83)	0.63	1.12 (0.48–2.64)	0.79
No surgery performed	9 (47)	1.74 (0.81–3.73)	0.16		
Indication SAT					
Certain relapse	24 (44)	2.27 (1.29–5.72)	0.01	2.04 (0.89–4.70)	0.09

The abbreviations used in the table are as follows: HR – hazard ratio; CI – confidence interval; SAT – suppressive antimicrobial therapy. ^*^ Reference.

### 3.2 Reported side effects

Of the 147 prescriptions, side effects were reported 36 times (24 %) by 31 individual patients (30 %). This led to a switch of antibiotic treatment 18 times in 12 patients (12 %) and cessation of antibiotics in 2 patients (2 %). Gastrointestinal side effects were most frequently reported ( 26/36 , 72 %), followed by rash ( 5/36 , 14 %), hepatitis ( 2/36 , 6 %), renal failure ( 1/36 , 3 %), tendinitis ( 1/36 , 3 %) and oral candidiasis ( 1/36 , 3 %). The frequency of side effects was not different between patients on low-dosage SAT and those on standard-dosage SAT ( p = 0.82). Detailed characteristics of the reported side effects per antimicrobial regimen are summarized in Table S7.

## 4 Discussion

In this cohort study, patients with OII treated with low-dosage SAT had a comparable outcome to patients treated with the standard dosage of antibiotics. The overall SAT success rate of 69 % is in line with comparable studies on SAT in OII that reported success rates between 59 % and 72 % (Prendki et al., 2017; Pradier et al., 2018; Escudero-Sanchez et al., 2020). To the best of our knowledge, this is the first study on OII focusing on the effectiveness of low-dosage SAT. Several studies on PJI included patients on lower-dosed SAT but did not compare its effectiveness with standard-dosage SAT (Siqueira et al., 2015; Bryan et al., 2017; Prendki et al., 2017; Wouthuyzen-Bakker et al., 2017; Leijtens et al., 2019).

Patients with OII on SAT represent a heterogenous group with a prognosis that is dependent on the timing of infection, the initial treatment and host factors. For the patients who were categorized in the “certain” relapse group (if SAT would have been withheld), we deemed it very unlikely that the infection was cured after initial treatment (i.e., patients treated with antibiotics only, late chronic infections treated with DAIR, and patients with a failure after the initial DAIR). In the “high-risk” group of patients, the risk of a relapse was considered to be substantial (if SAT would have been withheld) but not as high as in the certain group. The success rate of the high-risk group (81 %) was indeed higher than the certain group (56 %). It cannot be excluded that a proportion of patients within the high-risk group may have received SAT while their infection was already cured. This is a well-known uncertainty for all physicians who consider SAT for their patients with OII. Nonetheless, the outcomes of the survival analyses in this study suggest that low-dosage antibiotics could be a viable option for all patients on SAT, including patients whose infection will almost certainly relapse without suppression (Fig. 2c).

### 4.1 Duration of SAT

In many studies, SAT is recommended to be prescribed “indefinitely” (Marculescu et al., 2006; Byren et al., 2009; Prendki et al., 2017; Escudero-Sanchez et al., 2020). This is likely due to the uncertainty of whether chronic OII can ever be cured without implant removal and, if so, in which patients and within what time frame. Ideally, the duration of antibiotic treatment is based on the lifespan of the bacteria in the biofilm, but this lifespan is currently unknown. In our study, stopping SAT after 2 to 3 years resulted in a very low relapse rate, comparable to continuing SAT beyond 2 years. Moreover, 19 patients in our study were treated with antibiotics only (i.e., no surgical debridement or implant removal), and in 5 of those patients the discontinuation of SAT did not result in a relapse during 12 months follow-up. Our observation that a cure for OII may be achieved after a certain period of SAT has been reported before. Pavoni et al. (2004, n = 29) reported a cure rate of 66 % after stopping SAT within 1 year, but the indication for initiating SAT was not clearly defined in that paper. No relapses were reported by Bene et al. (2018) in 24 patients with acute PJI of the knee who stopped SAT after 20 months and a follow-up of 4 years. Pradier et al. (2018) reported 15 failures in 52 patients (29 %) on indefinite SAT compared to two relapses in 26 patients (8 %) in a historical cohort with a maximal duration of 2 years of SAT. Five other observational studies with a combined total of 120 patients reported sporadic SAT cessation after 6 to 36 months in 15 patients with one subsequent relapse (7 %) (Goulet et al., 1988; Segreti et al., 1998; Rao et al., 2003; Leijtens et al., 2019; Sandiford et al., 2019). Byren et al. (2009, n = 112) found a fourfold increase in relapse in the first 4 months after stopping SAT after a median time of 15 months in patients with PJI treated with DAIR; however, this occurred only in a minority of patients, and most of the patients were actually cured. In the study of Shah et al. (2020, n = 108), which included patients with knee PJI managed by DAIR, extending SAT beyond 12 months did not result in better outcome. The majority of relapses in our cohort occurred in the first 2 years during treatment with SAT, which is consistent with other studies (Prendki et al., 2017; Weston et al., 2018; Leijtens et al., 2019; Escudero-Sanchez et al., 2020). The relapse rate in the group of patients on SAT beyond 2 years was only 9 % in our study. In the largest cohort on SAT in PJI published to date ( n = 302), 33 % of failures were a microbiologically confirmed relapse and only 17 % of relapses could be attributed to the cessation of SAT (Escudero-Sanchez et al., 2020). The discrepancy in causative microorganisms between the initial OII and the subsequent relapse raises the question of whether most late failures on SAT actually stem from new infections with microorganisms that SAT could not have prevented. Further, a substantial part of SAT failures were culture negative. This suggests that the infection was cured but SAT could not prevent loosening of the implant.

In short, the assumption that every patient with OII and an indication for SAT needs to be treated indefinitely can be challenged: some patients might have been cured already before initiation of SAT, and for a subgroup of patients with an apparent “incurable” infection, 2 to 3 years of treatment with SAT (or perhaps even shorter) seems sufficient in the case of a favorable clinical course. To reliably assess and decide in whom and when SAT can safely be stopped, it is essential to use a personalized approach for each individual patient on suppression.

### 4.2 Concept of SAT

SAT is usually preceded by surgical debridement and a therapeutic antimicrobial phase with the goal to eradicate all metabolically active bacteria in the soft tissue, surrounding bone and biofilm. For this goal, dosing is based on pharmacokinetic and pharmacodynamic (PK/PD) indices for optimal effectiveness (Onufrak et al., 2016). After this phase, eventual remaining biofilm still contains metabolically inactive, dormant bacteria, so-called persisters, against which antibiotics are not effective (McConoughey et al., 2014). The only purpose of SAT is to kill those bacteria that switch back from a dormant state to metabolically active bacteria capable of causing a relapse of the infection. Within this concept, it is probable that the PK/PD targets for SAT are lower than for therapeutic treatment, and the results of this study with lower-dosed antibiotics support this hypothesis.

**Figure 3:**
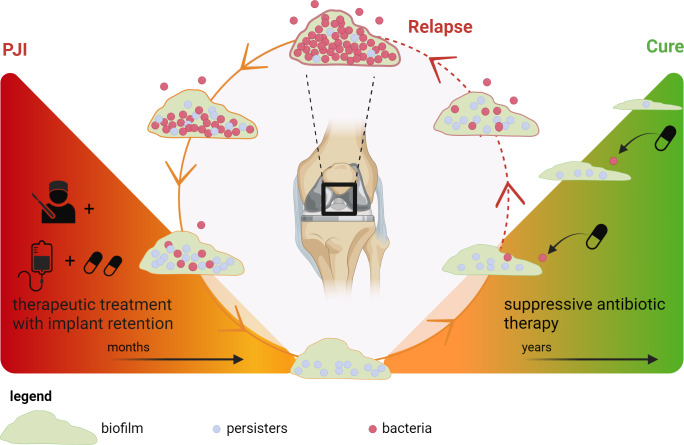
Concept of biofilm development during suppressive antimicrobial treatment in prosthetic joint infection. Left triangle: treatment of a chronic prosthetic joint infection with debridement and implant retention followed by therapeutic antimicrobial treatment. During this phase, all metabolically active bacteria in the (peri)prosthetic tissue are killed, but some persisters endure in the biofilm. Right triangle: suppressive antimicrobial therapy (SAT) is only aimed at those bacteria that switch back from a persister state to a metabolically active state, thereby preventing spread into the periprosthetic tissue which otherwise would lead to clinical relapse. For this specific goal, low-dosage SAT could be sufficient. Under these conditions, the biofilm slowly degrades and a cure can be achieved. Created with https://Biorender.com (last access: 15 January 2024).

Some patients with chronic OII were cured without implant removal. This suggests that degradation of the biofilm matrix on these implants and/or eradication of all bacteria including persisters can occur during SAT. This conceptual model of SAT is illustrated in Fig. 3.

Currently, the exact pathophysiology and duration of such a degradation process are unknown, and data on biofilm durability in OII while being treated with long-term antibiotics are absent. More insight into the pathophysiology of biofilms is needed to relate the strategy of stopping SAT after a set amount of time as well as lower (or less frequently) dosing of SAT to treatment success.

### 4.3 Suppressive treatment for FRI and SII 

This cohort contains the largest group of FRI treated with SAT to date. Only one small observational study reported on SAT for patients with FRI ( n = 5) (Ceccarelli et al., 2023). Data on SAT in SII are also scarce with the largest cohort reporting a 2-year survival free of treatment failure of 71 % in acute SII treated with DAIR followed by SAT compared to 33 % in those with acute SII treated with DAIR without SAT (Kowalski et al., 2007). The failure rate in our study was 21 % for SII. Although not statistically significant (likely due to the small sample size), PJI was associated more strongly with failure than FRI and SII. Although the pathophysiological concept of biofilm formation and treatment is the same for all OII, differences in local anatomy, condition of soft tissue, type of bone and foreign body material might influence clinical outcomes. More data are needed to understand the similarities and differences in these infections.

### 4.4 Antibiotics used

Beta-lactams and clindamycin were the most commonly used antibiotics in this study. Effectiveness and side effects were similar for the different antibiotics used in this study and consistent with other research (Siqueira et al., 2015; Leijtens et al., 2019; Escudero-Sanchez et al., 2020). Lower dosing did not result in less side effects. Perhaps even lower dosages are needed to reduce toxicity of these drugs. When choosing a drug for chronic antimicrobial therapy, side effects, drug–drug interactions and dosing frequency should be taken into account. Once daily regimens may improve medication adherence, but this has not been studied in SAT (Coleman et al., 2012; Weeda et al., 2016). Expert-opinion-based dosing schedules for SAT in OII from our institution are provided in Table 5.

**Table 5:** Expert-opinion-based dosing schedules for suppressive antimicrobial therapy in orthopedic implant infections.

Drug	Dosing
Amoxicillin	500 mg b.i.d.
Flucloxacillin	1000 mg b.i.d.
Amoxicillin / clavulanic avid	625 mg b.i.d.
Ciprofloxacin and levofloxacin	500 mg q.d.
Clindamycin	600 mg b.i.d.
Trimethoprim / sulfamethoxazole	960 mg q.d.
Tetracyclines	100 mg q.d.
Linezolid	300 mg q.d.

The abbreviations used in the table are as follows: q.d. – once daily; b.i.d. – twice daily.

### 4.5 Strengths and limitations

This study evaluated the effectiveness and side effects of well-specified different antimicrobial dosing strategies for SAT. Furthermore, this study included the largest series of patients with FRI treated with SAT. By including patients with a relatively short minimum follow-up duration, we reduced the possibility of missing early failures.

The study has several limitations, e.g. due to its retrospective design and selection bias, and confounding cannot be fully excluded. Adverse effects were not documented in a uniform manner. The study population is heterogenous regarding outcome because SII an FRI might have better prognosis than PJI. The relatively limited size of our cohort necessitates prospective data to validate these results. Lastly and most importantly, confirmative parameters which can discern cured patients from patients with a persistent biofilm do not exist. This likely has resulted in patients receiving SAT while the infection was already cured after initial management as discussed above. 

## 5 Conclusions

Based on this study, lower and/or less frequently dosed antibiotics may be a safe treatment option for patients with OII who have an indication for chronic suppressive therapy. Furthermore, stopping SAT after 2 to 3 years may be justified in patients who are clinically stable. This decision needs to be weighed for each individual patient. More research is needed to evaluate which patients really need SAT after the initial treatment phase. Further, larger cohort studies are warranted to confirm and validate the findings of this study to determine optimal dosing and duration of SAT and to identify an optimal set of clinical criteria for safe discontinuation.

## Supplement

10.5194/jbji-9-149-2024-supplement The supplement related to this article is available online at: https://doi.org/10.5194/jbji-9-149-2024-supplement.

## Data Availability

All raw data can be provided upon reasonable request from the corresponding author. Data are located in a controlled access data storage at Leiden University Medical Center.
