# Supplementary material for: Dosing and treatment duration of suppressive antimicrobial therapy in orthopedic implant infections: a cohort study
Source: J Bone Jt Infect. 2024 Jun 4;9(3):149–59. doi: 10.5194/jbji-9-149-2024 (PMC11187703; doi:10.5194/jbji-9-149-2024)
Supplement: The supplement related to this article is available online at: https://doi.org/10.5194/jbji-9-149-2024-supplement. [file jbji-9-149-supplement.pdf]

Supplement of J. Bone Joint Infect., 9, 149–159, 2024
https://doi.org/10.5194/jbji-9-149-2024-supplement
© Author(s) 2024. CC BY 4.0 License.

Supplement of

Dosing and treatment duration of suppressive antimicrobial therapy in orthopedic implant infections: a cohort study

Jaap L. J. Hanssen et al.

Correspondence to: Jaap L. J. Hanssen (j.l.j.hanssen@lumc.nl)

The copyright of individual parts of the supplement might differ from the article licence.

Table S1 diagnostic criteria of the 19 patients that did not receive surgery for the orthopedic implant infection.

Infection	Diagnosis[a]	Diagnostic criteria[a]	Microorganism
FRI upper arm	confirmed	Fistula, positive culture from aspiration	*Cutibacterium acnes*
PJI hip	likely	fever, wound healing history, CRP >10mg/l, positive culture from aspiration	*Streptococcus agalactiae*
PJI hip	likely	fever, wound healing history, CRP >10mg/l, positive culture from aspiration	*Staphylococcus epidermidis*
PJI hip	likely	Fever, purulence prosthesis, CRP >10mg, positive culture from aspiration	Group B *Streptococci*
FRI leg	confirmed	Fistula, positive culture from aspiration,	*Cutibacterium acnes*
PJI knee	likely	Radiological loosening, CRP >10mg/ml, positive culture from aspiration	*Staphylococcus haemolyticus*
FRI pelvis	suggestive	Bacteremia, fever, pain, PET suggestive of infection	*Staphylococcus aureus*
PJI shoulder	likely	Fever, pain, CRP>10mg/L, PET suggestive of infection, positive culture from aspiration	*Cutibacterium C. acnes*
PJI hip	likely	Fever, pain, CRP>10mg/L, ultrasound suggestive of infection, positive culture from aspiration	*Staphylococcus aureus*
PJI hip	likely	Fever, pain, CRP>10mg/L, ultrasound suggestive of infection, positive culture from aspiration	*Staphylococcus aureus*
FRI elbow	suggestive	Pain, redness, CRP>10mg/L, ultrasound suggestive of infection, positive culture from aspiration	*Staphylococcus epidermidis*
SII	suggestive	Pain, fever, CRP>10mg/L, CT suggestive of infection, positive culture from CT guided aspiration	*Staphylococcus aureus*
PJI knee	suggestive	Fever, pain, redness, previous wound healing problems CRP>10mg/L, positive culture from aspiration	*Staphylococcus aureus*
PJI hip	confirmed	>2 intraoperative cultures	*Staphylococcus haemolyticus*
PJI knee	suggestive	Recent bacteremia, fever, redness, CRP>10mg/L, positive culture from aspiration	*Streptococcus oralis*
PJI shoulder	suggestive	Redness, radiological signs of loosening, CRP>10mg/L, positive culture from aspiration	*Cutibacterium acnes*
PJI shoulder	suggestive	Fever, redness, CRP>10mg/L, positive culture from aspiration (pus)	*Cutibacterium acnes*
PJI elbow	confirmed	Fistula, positive culture from aspiration	*Enterobacter cloacae*
PJI knee	suggestive	Redness, pain, radiological loosening, ultrasound suggestive of infection, positive culture from aspiration (pus)	*Staphylococcus epidermidis*

The abbreviations used in the table are as follows: FRI – fracture related infection; PJI - prosthetic joint infection; SII – spinal implant infection. [a] according to EBJIS 2021 criteria for PJI and the AO Foundation and EBJIS 2020 consensus definition for FRI.

Table S2 Characteristics of the antibiotic prescribed for suppressive antimicrobial treatment (SAT) in 108 patients with an orthopaedic implant infection

Antibiotic	Number of prescriptions (n, %)	Daily dosage (n)	Duration SAT in month mean (range)	Failure (n, %)
Flucloxacillin	26	1000 mg q.i.d. (3)<br>1000 mg t.i.d. (3)<br>1000 mg b.i.d. (12)<br>500 mg q.i.d. (1)<br>500 mg t.i.d. (1)<br>500 mg b.i.d. (6)	11 (1-91)	7 (27)
Amoxicillin	25	1000 mg q.i.d. (3)<br>1000 mg t.i.d. (1)<br>1000 mg b.i.d. (10)<br>500 mg q.i.d. (2)<br>500 mg t.i.d. (1)<br>500 mg b.i.d. (7)<br>750 mg b.i.d. (1)	18 (1-81)	3 (12)
Amoxicillin/clavulanic acid	6	1250 mg t.i.d. (1)<br>1250 mg b.i.d. (1)<br>625 mg b.i.d. (4)	7 (1-26)	2 (33)
Feneticillin	3	500 mg q.i.d. (2)<br>500 mg b.i.d. (1)	32 (20-41)	2 (66)
Clindamycin	31	600 mg t.i.d. (3)<br>600 mg b.i.d. (20)<br>300 mg b.i.d. (5)<br>300 mg t.i.d. (4)	18 (1-117)	7 (22)
Sulfamethoxazole/trimethoprim	18	960 mg b.i.d. (5)<br>960 mg q.d. (11)<br>480 mg q.d. (2)	16 (4-62)	4 (22)
Doxycycline	14	100 mg q.d. (14)	23 (5-62)	2 (14)
Ciprofloxacin	9	750 mg b.i.d. (4)<br>500 mg b.i.d. (1)<br>750 mg q.d. (3)<br>500 mg q.d. (1)	23 (1-69)	3 (30)
Levofloxacin	5	500 mg q.d. (4)<br>250mg q.d. (1)	22 (1-33)	1 (20)
Moxifloxacin	3	400 mg q.d. (3)	10 (4-92)	0
Linezolid	3	600 mg q.d. (1)<br>300 mg q.d. (1)<br>150 mg q.d. (1)	11 (9-33)	1 (33)
Rifampicin	2	300 mg q.d. (2)	21-22	1 (50)
Fluconazole	3	200 mg q.d. (3)	17 (1-117)	1 (33)

The abbreviations used in the table are as follows: q.d. - once daily; b.i.d. - twice daily; t.i.d. - three times a day; q.i.d. - four times a day.

Table S3 Microbiological characteristics of the index pathogens of the 34 patients with failure on suppressive antimicrobial treatment (SAT)

	All failures (%)	Relapse with index pathogen	Development of SAT resistance	New infection with different pathogen	Culture negative	No tissue for cultures obtained
Number of patients (%)	34 (100)	11 (32)	4 (12)	9 (26)	7 (21)	7 (21)
Index pathogen						
Staphylococcus aureus	8 (24)	2	1	3	1	2
Coagulase negative staphylococci	14 (41)	4	2	4	4	2
S. epidermidis	11	4	2	3	3	1
S. hemolyticus	2			1		1
S. capitis	1				1	
Gram negative species	12 (35)	4		5	2	1
Pseudomonas aeruginosa	4 (9)	1		2	1	
E. coli	1			1		
Acinetobacter baumanii	1			1		
Proteus mirabilis	3	2				1
Enterobacter cloacae	3	1		1	1	
Enterococci	8 (24)	2		3	2	1
E. faecalis	6	1		3	1	1
E. faecium	2	1			1	
Streptococci	3 (9)	1	0		2	
S. agalactiae	1				1	
Beta-hemolytic streptococci	2	1			1	
Cutibacterium acnes	5 (6)	1	1		1	2
Candida albicans	2 (6)			1	1	
Bacteroides fragilis	1 (3)	1				
Corynebacterium species	1 (3)			1		
Polymicrobial	13 (38)	3		4	5	1

Table S4 Microbiological characteristics of the 9 patients with failure with a new pathogen cultured

Index pathogen(s)	New pathogens(s)
Staphylococcus aureus, Pseudomonas aeruginosa, Enterococcus faecalis	*Stenotrophomonas maltophilia, Achromobacter xylosoxidans, Enterococcus faecium, Candida albicans*
Staphylococcus haemolyticus, Pseudomonas aeruginosa, Enterococcus faecalis	*Staphylococcus epidermidis*
Staphylococcus epidermidis	*Staphylococcus epidermidis,* Peptoniphilus species*, Prevotella melaninogenica*
Staphylococcus aureus	*Enterococcus faecalis,* alpha hemolytic streptococci
Staphylococcus epidermidis, Enterococcus faecalis, Escherichia coli	*Morganelli morgani, Bacteroides fragilis, Pseudomonas aeruginosa, Staphylococcus epidermidis*
Staphylococcus aureus	*Enterococcus faecalis, Escherichia coli, Klebsiella pneumoniae, Proteus vulgaris*
Candida albicans	*Enterococcus faecalis, Staphylococcus aureus*
Staphylococcus epidermidis, Acinetobacter baumanii, Corynebacterium species	Anaerococcus species
Enterobacter cloacae	*Streptococcus agalactiae*

Table S5 Time to failure in 4 patients with development of resistance against suppressive antimicrobial treatment

Index pathogen	Antibiotic treatment	months of treatment till relapse
Staphylococcus aureus[a]	flucloxacillin 1000 mg b.i.d.	15
Staphylococcus epidermidis	doxycycline 100 mg q.d.	9
Staphylococcus epidermidis	flucloxacillin 500 mg t.i.d.	11
Cutibacterium acnes	clindamycin 300 mg b.i.d.	22

[a] cultures of relapse showed a borderline resistant *Staphylococcus aureus* (BORSA) with a MIC for oxacillin of 6 mg/L.

Table S6 Analysis of clinical characteristics potentially associated with failure of suppressive antimicrobial therapy for patient with PJI (n=67)

	Failure n (%)	Univariable analysis HR (95% CI)	p-value	Multivariable analysis HR (95% CI)	p-value
Patient factors					
Age >70	13 (43)	1.31 (0.61–2.79)	0.49		
Smoker	5 (50)	2.06 (0.77–5.51)	0.15		
Charlson comorbidity index >2	19 (37)	0.67 (0.29–1.55)	0.35		
Diabetes mellitus	4 (67)	4.08 (1.38–12.052)	0.01	4.41 (1.41–13.76)	0.01
Previous PJI	16 (49)	1.34 (0.62–2.89)	0.45		
Revised prosthesis	18 (51)	1.47 (0.66–3.27)	0.35		
Chronic PJI	18 (46)	1.46 (0.66–3.25)	0.35		
Tumor endoprosthesis	15 (40)	0.99 (0.46–2.11)	0.97		
Anatomic location					
Hip	8 (25)	1			
Knee	13 (48)	2.29 (0.95–5.54)	0.07		
Upper limb	6 (75)	6.21 (2.07–18.59)	0.01	3.95 (1.43–10.92)	0.01
Microbiology					
Staphylococcus aureus	5 (33)	0.86 (0.32–2.27)	0.76		
Coagulase negative staphylococci	11 (44)	1.13 (0.52–2.44)	0.76		
Streptococci	3 (20)	0.29 (0.09–0.98)	0.05	0.42 (0.12–1.45)	0.17
Enterococci	5 (39)	1.12 (0.42–2.95)	0.82		
Gram-negatives	6 (50)	1.33 (0.54–3.31)	0.54		
Polymicrobial infection	13 (31)	1.03 (0.46–2.31)	0.93		
Clinical aspects					
<12weeks antibiotic treatment before SAT	17 (40)	1.12 (0.51–2.42)	0.80		
C-Reactive protein at start SAT >=20	10 (46)	1.87 (0.82–4.30)	0.14		
Low dosage SAT	21 (45)	1.26 (0.51–3.13)	0.62	1.05 (0.41–2.69)	0.92
No surgery performed	7 (50)	1.45 (0.61–3.45)	0.40		
Indication SAT					
"certain failure"	21 (48)	2.10 (0.84–5.25)	0.11	1.89 (0.71–5.02)	1.89

The abbreviations used in the table are as follows: HR – hazard ratio; CI – confidence interval; SAT – suppressive antimicrobial therapy. [a] Reference

Table S7 Reported side effects of suppressive antimicrobial treatment per antibiotic

Antibiotic	Side effects (n)	Switch or stop SAT (n, %)
Flucloxacillin	GI (4)	4 (15)
Amoxicillin	GI (8)	3 (12)
	skin (1)	
Amoxicillin/clavulanic acid	GI (1)	0
	oral candida (1)	
Feneticillin	GI (2)	
Clindamycin	GI (9)	7 (22)
	skin (1)	
	liver (1)	
Sulfamethoxazole/trimethoprim	GI (1)	1 (6)
	Renal (1)	
Doxycycline	Skin (2)	2 (14)
Ciprofloxacin		0
Levofloxacin	Liver (1)	2 (40)
	Tendon (1)	
Moxifloxacin		0
Linezolid	Skin (1)	1 (33)
	GI (1)	
Rifampicin		0
Fluconazole	GI (1)	0

The abbreviations used in the table are as follows: SAT - suppressive antimicrobial treatment; GI - gastrointestinal.
